# An Atypical Presentation of Severe Dissecting Aortic Aneurysm: A Case Report and Literature Review

**DOI:** 10.7759/cureus.15752

**Published:** 2021-06-18

**Authors:** Ahmed Mowafy, Payal Rath, Ahmed Eladly, Ahmed Omran

**Affiliations:** 1 Internal Medicine, Rutgers/Trinitas Regional Medical Center, Elizabeth, USA; 2 Clinical Research, University of Louisville, Louisville, USA

**Keywords:** asymptomatic aortic dissection, aortic dissection diagnosis, aortic dissection management, literature review of disease, atypical presentation

## Abstract

A 55-year-old male patient with no past medical history presents to the ED to complain of exertional dyspnea and bilateral lower limb swelling. The patient was diagnosed with a severe dissecting aortic aneurysm on trans-thoracic echocardiography and later confirmed by a contrast CT aortogram. The patient was immediately started on anti-impulse therapy with labetalol infusion and underwent emergent aortic dissection repair. This case demonstrates an atypical presentation of a life-threatening condition that might have been missed on the initial assessment.

## Introduction

Aortic dissection is an uncommon (2.6-3.5 per 100,000 person-years), but morbid condition, with mortality rates, reported around 20-30% [1]. The most common risk factors for dissecting aortic aneurysms are uncontrolled hypertension, atherosclerosis, dyslipidemia, and smoking. Less common risk factors include connective tissue disorders like Marfan syndrome, vasculitis, and traumatic causes [2]. Patients are usually 60-80-year-old men (mean 63y). Women typically present later (mean 67y). It typically presents severely and catastrophically, with chest pain and hemodynamic instability being the most common onset symptoms. This case report demonstrates an atypical presentation that closely mimics that of decompensated congestive heart failure. However, the patient was ultimately diagnosed with a severe thoracic aortic dissection secondary to uncontrolled hypertension, with a paradoxically preserved cardiac efficiency.

## Case presentation

A 55-year-old male patient with no past medical history presented to the ED with complaints of shortness of breath, with exertion, then later at rest, progressive bilateral leg swelling, and generalized fatigue for the preceding two weeks. He endorsed orthopnea and nocturnal paroxysmal dyspnea but denied headache, dizziness, syncope, chest pain, cough, abdominal pain, nausea, vomiting, diarrhoea, constipation, claudication, varicose veins, and limb numbness.

On presentation, the patient was afebrile, body temperature 98.7, tachycardic and hypertensive, heart rate 103 bpm, BP 169/76 mmHg, respiration rate (RR) 20/min, O_^2^_ saturation 98% breathing ambient air. Physical examination revealed faint bilateral pulmonary crackles over the lower lung fields, in addition to a 3/6 harsh diastolic murmur auscultated over the left sternal border and apex, with a non-displaced point of maximal apical impulse. It was also noted that the patient had moderate to severe bilateral lower limb pitting edema up to the knees. The remainder of his examination was non-pertinent.

His labs revealed Hgb 10.7 gm/dL (12-16 gm/dL), WBC 8.1(4.8-10.8), serum sodium 138 mMol/L (136-146 mMol/L), potassium 4.1 mMol/L (3.6-5.1 mMol/L), chloride 107 mMol/L (101-111 mMol/L), bicarbonate 21 mMol/L (22-32 mMol/L), creatinine 0.8 mg/dL (0.4-1.0 mg/dL), blood urea nitrogen (BUN) 16 mg/dL (8-20 mg/dL), albumin 2.7 gm/dL (3.5-4.8 gm/dL), total Bilirubin 1.6 mg/dL (0.4-2.0 mg/dL), alanine aminotransferase (ALT) 48 U/L (17-63 U/L), aspartate aminotransferase (AST) 31 U/L (15-41 U/L).

His brain natriuretic peptide (BNP) was 15 pg/ml (<100 pg/ml). His lipid panel revealed a cholesterol of 160 mg/dL (120-200 mg/dL), high-density lipoproteins (HDL) 28 mg/dL (29-71 mg/dL), low-density lipoproteins (LDL) 115 mg/dL (<100 mg/dL), and triacyl glycerides 87 mg/dL (40-199 mg/dL). His initial Troponin I was 0.04 ng/ml (<0.5 ng/ml).

**Figure 1:**
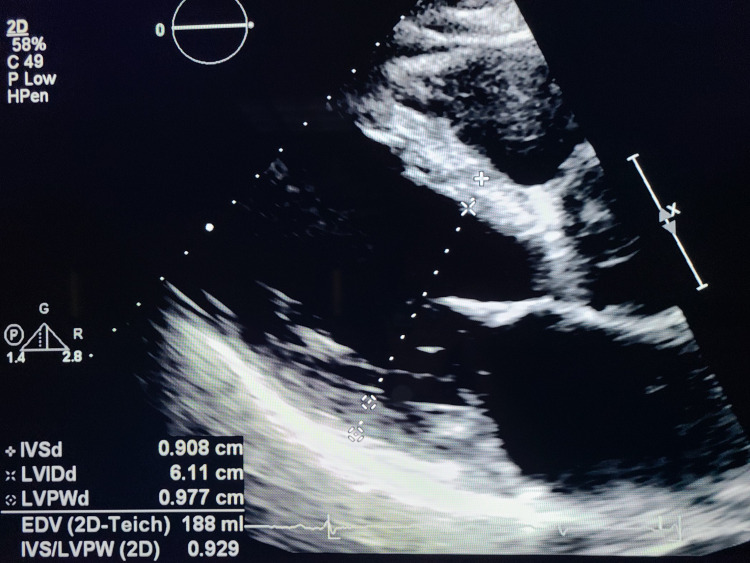
Echocardiography demonstrating aortic regurgitation

**Figure 2:**
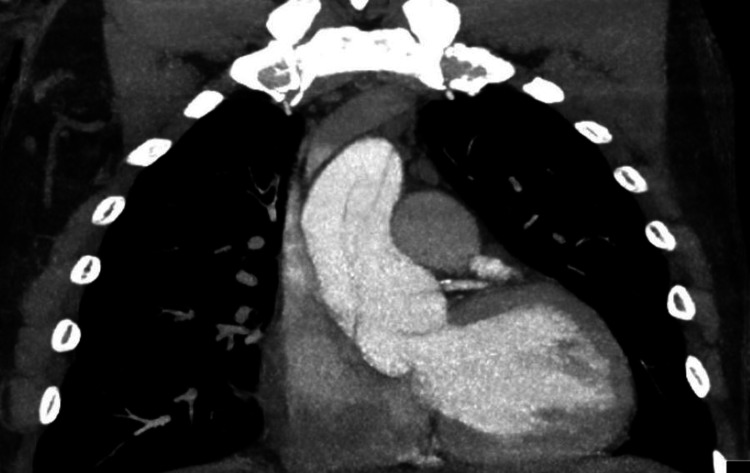
CT angiogram of the chest demonstrating aortic double lumen

A trans-thoracic echocardiogram was obtained, and it revealed the patient’s left ventricular ejection fraction to be 50-55%, with hypertensive cardiomyopathy and a globally decreased left ventricular systolic function. It also revealed a mild mitral valve regurgitant jet, and a mildly dilated pulmonary artery and an elevated pulmonary artery systolic pressure. But the most important finding was a moderate to severe aortic regurgitation, with Type I dissection of the ascending and descending aorta, and the aortic arch (figures 1-2).

**Figure 3:**
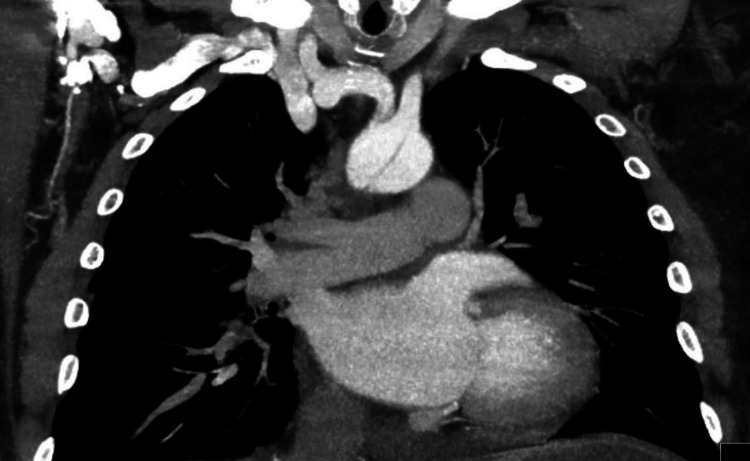
CT angiogram demonstrating aortic arch double lumen

**Figure 4:**
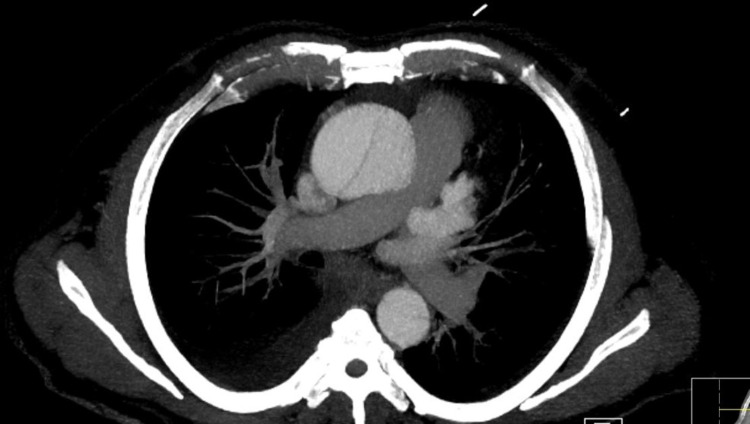
CT angiogram demonstrating descending aorta double lumen

**Figure 5:**
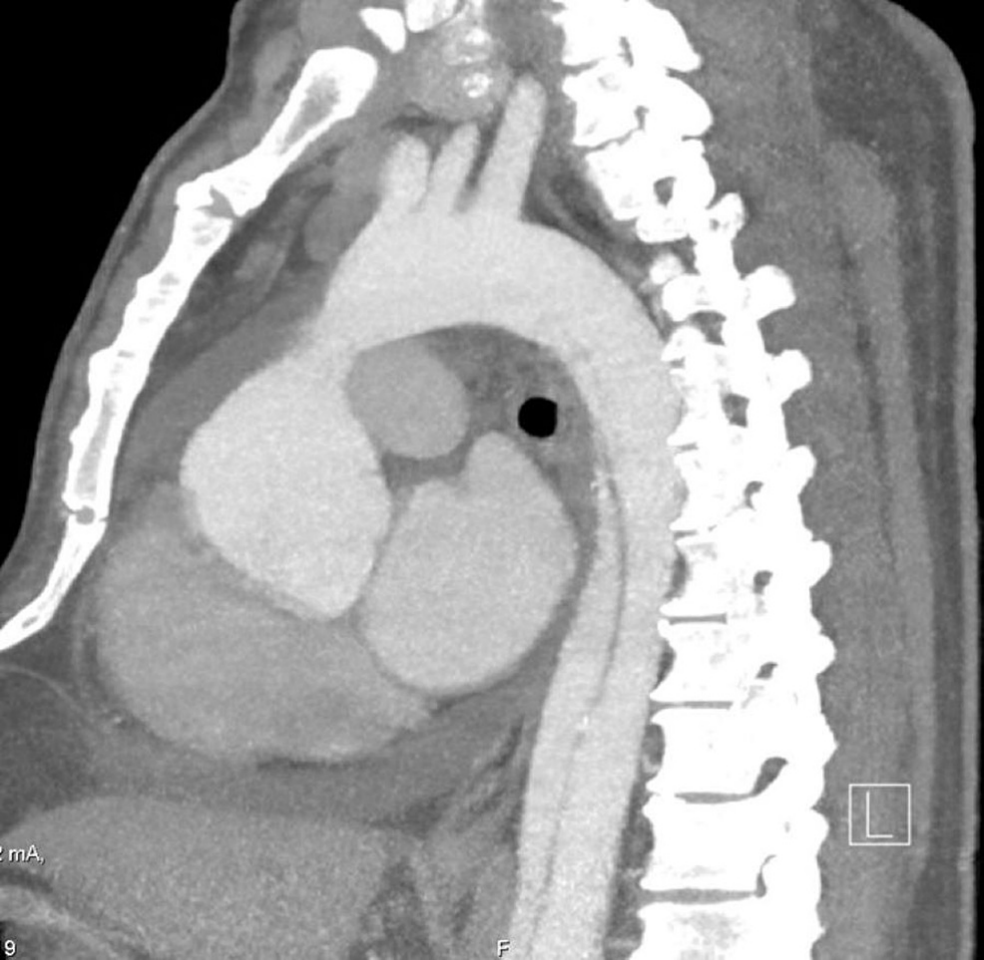
CT angiogram of the chest demonstrating the descending aorta double lumen

**Figure 6:**
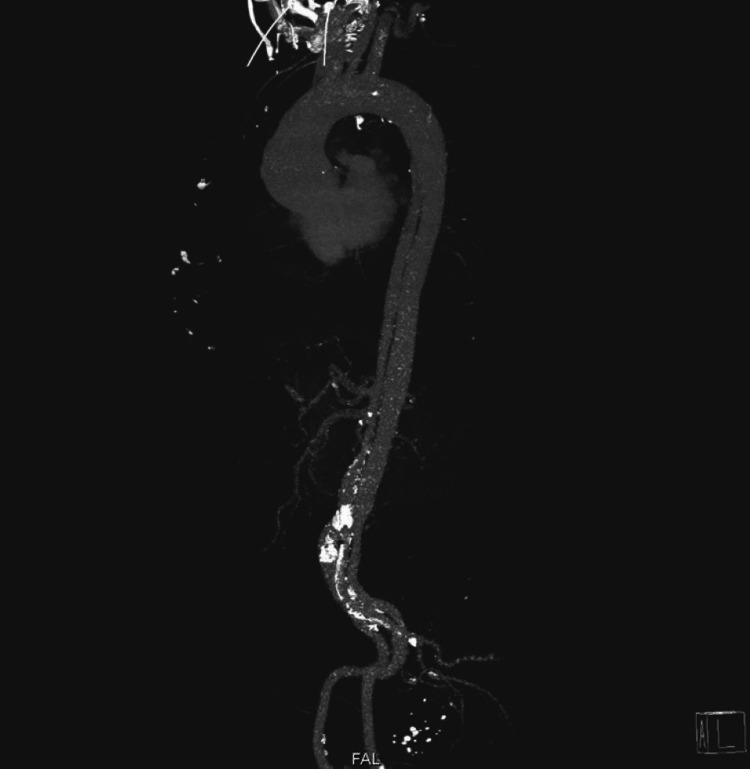
An aortogram demonstrating the extent of the dissection from the aortic root to the iliac bifurcation

Upon discovering these findings, a contrast CT angiogram of the chest and an aortogram was obtained, which in turn revealed severe aortic dissection extending from the aortic root all the way down to the iliac bifurcation (figures 3-6).

The patient was immediately transferred to the Intensive Care Unit, and started on furosemide for volume status optimization, and started on a labetalol drip for decreasing the blood pressure and ventricular contraction velocity to alleviate the shearing force on the aortic wall (anti-impulse therapy), with a target heart rate of less than 60 bpm and blood pressure less than 120/89 mmHg.

After stabilization, the patient was transferred to another facility and underwent a successful aneurysm surgical repair and aortic valve replacement. He was discharged on continuous anti-impulse therapy for outpatient follow up.

## Discussion

As stated before, aortic dissection is a life-threatening condition that usually presents acutely and with catastrophic outcomes. Mortality rates vary according to patients' age, gender, and stage at presentation. However, according to a whole nation study conducted on 153 patients by Melvinsdottir et al., around 17% died before hospital arrival. In contrast, the risk of death for patients who arrived alive at a hospital was 21.4% within 24h and 45.2% at 30 days [3]. The study also shows that with proper surgical or medical management, depending on the condition, the 30-day mortality rate improves with every year, and the five-year survival rate has been increasing steadily.

Aortic dissection can be classified according to a presentation of "acute" and "chronic", depending on the duration of symptoms before a presentation. Less than two weeks between the onset of symptoms and presentation falls into the acute category, and beyond that window, it is labelled a chronic condition. This classification is of significance given that acute onsets are much more liable to complications related to the extension of the dissection and involvement of major aortic branches like the carotids, left subclavian, or even coronaries.

Perhaps a more relevant classification regarding morbidity and mortality rates would be the Stanford classification. According to this classification, a Type A dissection involves the ascending aorta, and a Type B dissection does not. Unfortunately, Type A dissections are almost twice as common as Type B [2]. Moreover, Type A dissection management is generally surgical, while Type B is standardly managed medically.

Mortality in Type A cases managed surgically ranges between 18-22%, depending on postoperative complications. In comparison, mortality among Type A patients managed medically is 57%, a value that naturally explains the inclination to manage Type A surgically. Type B, on the other hand, shows mortality benefit when managed medically, as mortality rates among medically treated patients are around 12% within the first 30 days, while patient's who are managed with surgical or endovascular intervention experienced a mortality rate of 15-21% [4]. A study by Pape et al. in 2015 proposes a mortality rate of 30% in patients with Type B aneurysm who were treated surgically compared to a rate of 10% who were managed medically [5].

In our case, the most common presenting symptom for a dissecting aneurysm is sharp, severe chest pain, with occasional radiation to the back in >90% of patients, while painless dissections account for less than 6.5% of cases. Signs like hemodynamic instability and pulse and blood pressure disparity between upper limbs, which our patient did not have, present in around 33% of cases, correlate poorly with mortality and complication rates [2].

Aortic regurgitation is also a common sign in proximal dissections, occurring in 50% of cases [6]. Acute decompensated congestive heart failure is a known complication of severe aortic dissections. However, it typically coexists with syncope, chest pain, abdominal pain, and malperfusion symptoms to vital organs and distal peripheries [5].

Our patient had acute decompensated heart failure symptoms but none of the other symptoms that usually accompany it. An echocardiography revealed an LVEF of 50-55%, effectively ruling out reduced ejection fraction heart failure. Furthermore, his lab work did not reveal any organ affection, creatinine, and liver enzymes within normal limits.

The most likely etiology behind the development of our patient's condition is undiagnosed, uncontrolled hypertension. He was hypertensive on presentation and stated that he had never seen a physician in his life before. So our patient falls into the narrow fraction of cases with acute, severe dissecting aortic aneurysm with almost none of the typical presenting symptoms. And especially regarding the severity of the condition and the dissection involving the entire aorta from root to bifurcation, his quality of life before presentation and survival are remarkable.

## Conclusions

Severe aortic dissection is a severe and often life-threatening condition, and clinicians are often comfortable with its diagnosis and management, given how drastically it typically presents. However, in this case, we demonstrate a severe case of aortic dissection that presented with almost none of the typical markers of the condition, especially the severity. We hope this case report would raise the medical community's awareness regarding the rare subtle cases that could be missed or misdiagnosed because of atypical presentations.
